# Public health implications of multidrug-resistant and methicillin-resistant *Staphylococcus aureus* in retail oysters

**DOI:** 10.1038/s41598-025-88743-5

**Published:** 2025-02-06

**Authors:** Rahma Mohammed, Sara M. Nader, Dalia A. Hamza, Maha A. Sabry

**Affiliations:** Department of Zoonoses, Faculty of Veterinary Medicine, Cairo University, PO Box 12211, Giza, Egypt

**Keywords:** *S. aureus*, MRSA, Multi-drug resistance, Virulence factors, Oysters, Egypt, Microbiology, Molecular biology

## Abstract

Methicillin-resistant *Staphylococcus aureus* (MRSA) is a major challenge for food safety and public health. This study aimed to investigate the potential role of oysters sold in Egypt as a source for *Staphylococcus aureus* (*S. aureus*), MRSA, and multidrug-resistant methicillin-resistant *Staphylococcus aureus* (MDR-MRSA). It also examined the occurrence of *tsst-1* virulence gene and assessed the associated zoonotic risks. Thirty-three pooled fresh oyster samples were acquired from different retail fish markets in Egypt. *S. aureus* was identified by conventional culture-based and molecular methods. Antimicrobial resistance was performed by the disk-diffusion method, and the multiple antibiotic resistance index (MARI) was calculated. Antimicrobial resistance (*mecA* and *mecC*) and virulence (*tsst-1*) genes were screened using polymerase chain reaction. The clustering of virulent MDR-MRSA isolates was performed using R with the pheatmap package. The prevalence of *S. aureus* was 39.4% (13 /33), and 77% of them (10/13) were classified as MDR with MARI values greater than 0.2. Notably, 46.2% (6 /13) of isolates were identified as MRSA and all MRSA isolates displayed MDR. Of the MDR-MRSA isolates, 66.7% (4 /6) possessed the *mecA* gene, while 16.7% (1 /6) tested positive for the *mecC* gene. Additionally, the *tsst-1* gene was identified in one isolate (16.7%). Interestingly, two MDR-MRSA isolates exhibited a clustered pattern. The study sheds light on the emergence of virulent MDR-MRSA isolates in Egyptian oysters. It highlights oysters as a potential source for spreading these isolates within aquatic ecosystems, posing a threat to food safety and public health.

**Supplementary Information:**

The online version contains supplementary material available at 10.1038/s41598-025-88743-5.

## Introduction

Antimicrobial resistance (AMR) is currently one of the major global issues.  Millions of deaths, permanent disabilities, and increased medical costs are the results. It also endangers food safety and causes deaths among humans and animals^1–3^. The misuse and overuse of antibiotics, whether in human or veterinary medicine, are directly related to the emergence of resistant bacterial strains^4^.

*Staphylococcus aureus*, a superbug, is accountable for a wide spectrum of hospital and community-acquired infections^5^. Additionally, it is a foremost contributor to foodborne illnesses, particularly foodborne intoxications^6^.

Over time, the majority of *S. aureus *strains have developed resistance to β-lactam antibiotics, leading to the emergence of MRSA^7^. One of the key factors contributing to β-lactam resistance in MRSA is the presence of a highly transmissible mobile genetic element-staphylococcal cassette chromosome mec (SCCmec) which harbors *mecA* and its analog *mecC *genes encoding penicillin-binding proteins PBP-2 A and PBP-2 C, respectively^8^. These genes provide resistance to most β-lactams, by interfering with the drug’s ability to bind to cell wall proteins^9^.

Indeed, MRSA has become a focal point of public health concern and a potentially lethal pathogen^10^. Beyond β-lactams, MRSA exhibits resistance to a range of other antibiotics, including macrolides, tetracycline, aminoglycosides, chloramphenicol, lincosamides, and fluoroquinolones^11,12^. This multi-drug resistance complicates MRSA treatment and allows the bacteria to survive in environments where antibiotic selection pressure exists^5,13^.

The pathogenicity of *S. aureus *primarily stems from the production of potent super-antigenic toxins, such as toxic shock syndrome toxin-1 (TSST-1), which incriminated in toxic shock syndrome (TSS) in humans^14^. Toxic shock syndrome is a rare, life-threatening, multisystemic disease characterized by rapid onset of fever, erythematous skin rash, hypotension, hemodynamic shock, multiorgan failure, and death^15–17^.

Owing to the genomic plasticity of bacteria, several virulent and antimicrobial-resistant strains of *S. aureus *have evolved primarily due to horizontal gene transfer and insurgence of chromosomal point mutations^18,19^. Understanding the genetic characteristics and virulence factors of these strains is crucial for effective infection control and management.

MRSA has the ability to colonize and infect a wide range of hosts, existing in distinct ecological niches^20,21^. This bacterium can contaminate animal-derived food products and seafood throughout the production chain, from farm to table^22–24^. Additionally, MRSA is frequently found in human sources globally, emphasizing its zoonotic potential and the role of the food production chain as a conduit for transmission of these resistant strains between humans and animals^25–27^.

Bivalve mollusks, including oysters, have a remarkable filtering capacity with low selectivity, leading them to accumulate biological contaminants such as *S. aureus*^24,28–30^. This accumulation represents a significant risk to consumers, especially when consuming raw or undercooked oysters^31^.

Egypt has been identified as a hyperendemic Mediterranean country for MRSA, exhibiting heightened levels of MRSA among *S. aureus *clinical isolates^32,33^. Given the significant concern regarding zoonotic MRSA transmission, it is crucial to assess the occurrence of *S. aureus*, MRSA, and MDR-MRSA in oysters sold in Egypt. Additionally, investigating the *tsst-1* virulence gene profile is essential for a comprehensive understanding of the potential risks associated with this pathogen.

## Methods

### Ethical approval

The protocol was reviewed and approved by the Institutional Animal Care and Use Committee (IACUC) of the Faculty of Veterinary Medicine, Cairo University, Egypt (Vet CU18042024932).

### Samples collection and processing

A total of 330 fresh oysters were randomly collected from different retail fish markets in Cairo and Giza governorates over one year, from December 2021 to December 2022. The samples were immediately transported to the laboratory under sterile refrigerated conditions and divided into 33 pools containing ten oyster samples. Each pool corresponds to a distinct market, emphasizing one per market.

The external valves of oysters were thoroughly washed with sterile water and aseptically opened. The digestive tissues were dissected, cleaned, and finely chopped to a paste-like consistency to ensure the uniformity of the starting material^34^.

### Isolation and identification of *S. aureus*

Aliquots of 2 g from each pool were enriched overnight in 5 ml Brain heart infusion broth (Oxoid, Hampshire, UK) before plating on mannitol salt agar medium (Oxoid, Hampshire, UK) and incubated aerobically at 37 °C for 24 h. Suspected *S. aureus* colonies were sub-cultured to obtain a pure culture and were examined for colony morphology, Gram staining, standard biochemical tests, and coagulase test according to Quinn et al.^35^and Mahon and Lehman^36^.

To prevent bacterial contamination and spread in the laboratory, several stringent measures were implemented, including the use of personal protective equipment (PPE), adherence to strict hand hygiene protocols, regular disinfection of work surfaces and equipment, and proper disposal of biological wastes.

### Molecular identification of *Staphylococcus* Genus and the species *S. aureus*

Genomic DNA was extracted from isolates using the boiling method^37^. All *S. aureus* isolates were molecularly confirmed by PCR with *Staphylococcus* 16S rRNA primers to confirm the *Staphylococcus* genus according to Zhang et al.^38^, and with the *nuc* gene to confirm the species *S. aureus* according to McClure et al.^39^. The reaction mixtures were carried out on a total volume of 25 µl, containing 3 µl of template DNA from each isolate, 12.5 µl of Emerald Amp MAX PCR master mix (Takara, Japan), 0.5 µl of each primer (10 pmol/µl; Metabion, Germany) and completed up to 25 µl by PCR-grade water. The PCR amplicons were electrophoresed on a 1.5% agarose gel and visualized under ultraviolet light. The specific oligonucleotide primers set, and amplification conditions are displayed in Table 1.

**Table 1:** The sequence of oligonucleotide primers used for PCR amplification of the *Staphylococcus *16S rRNA, *S. aureus* *nuc* gene, *mecA* and *mecC* genes and *tsst-1*  virulence gene.

Gene (bp)	Primer sequence (5ʹ–3ʹ)	Cycling Conditions	Reference
**16S rRNA** (756 bp)	**F**: AAC TCT GTT ATT AGG GAA GAACA **R**: CCA CCT TCCTCC GGT TTG TCA CC	94 ºC ,5 min; 30 cycles (94 ºC, 1 min; 50 ºC , 1 min ; 72 ºC, 1 min), 72 ºC ,5 min	^38^
***nuc*** (279 bp)	**F**: GCGATTGATGGTGATACGGTT **R**: AGCCAAGCCTTGACGAACTAAAGC	95 ºC ,5 min; 30 cycles (95 ºC, 30 s; 55 ºC , 30 s ; 72 ºC, 1 min), 72 ºC ,10 min	^39^
***mecA*** (162 bp)	**F**:TCCAGATTACAACTTCACCAGG **R**:CCACTTCATATCTTGTAACG	94 ºC ,5 min; 32 cycles (94 ºC, 35 s; 54 ºC , 1 min; 72 ºC, 35 s), 72 ºC ,7 min	^46^
***mecC*** (138 bp)	**F**:GAAAAAAAGGCTTAGAACGCCTC **R**: GAAGATCTTTTCCGTTTTCAGC		
***tsst-1*** **(398 bp)**	**F**: TTATCGTAAGCCCTTTGTTG **R**: TAAAGGTAGTTCTATTGGAGTAGG	94 ºC ,5 min; 40 cycles (94 ºC, 40 s; 60 ºC , 40 s ; 72 ºC, 1 min), 72 ºC ,5 min.	^47﻿^

### Antimicrobial susceptibility testing (AST)

Antimicrobial susceptibility patterns of all confirmed *S. aureus *isolates were determined using the Kirby-Bauer disk diffusion method on Mueller Hinton Agar (MHA) (HiMedia) following Clinical and Laboratory Standards Institute (CLSI) guidelines^40^.

Fourteen antibiotics commonly prescribed in human and animals^41,42^ (Oxoid, Hampshire, UK) were used, representing nine different antimicrobial classes: β-lactams (Penicillins: ampicillin 10 µg, methicillin 5 µg, Cephalosporins: cefoxitin 30 µg), Aminoglycosides (amikacin 30 µg and gentamycin 10 µg), Fluorquinolones (ciprofloxacin 5 µg and levofloxacin 5 µg), Macrolides (erythromycin 30 µg and azithromycin 15 µg), Tetracycline (doxycycline 30 µg), Sulfonamides (trimethoprim/sulfamethoxazole 1.25 µg/23.75 µg), Phenicols (chloramphenicol 30 µg), lincosamide (clindamycin 2 µg), Ansamycins (rifampicin 5 µg).

*Staphylococcus aureus* isolates that test resistant to cefoxitin should be reported as MRSA according to CLSI^40^. Additionally, MDR isolates were defined as those not susceptible to at least one antimicrobial agent in three or more antimicrobial classes^43^.

The MARI is calculated by dividing the number of antibiotics to which the organism is resistant by the total number of antibiotics tested^44^.The MARI value greater than 0.2 indicates that the isolate originated from a source where antibiotics were used extensively and/or in large quantities, while a MARI of 1.0 signifies that the isolate is resistant to all antibiotics tested^45^.

### Molecular confirmation of MRSA

Isolates exhibiting phenotypic resistance to cefoxitin underwent additional confirmation through Multiplex PCR detection of the methicillin resistance-encoding genes, *mecA*, and *mecC*, following the protocols outlined by Doğan et al.^46^. The reaction mixtures were carried out on a total volume of 25 µl, containing 5 µl of template DNA from each isolate, 12.5 µl of Emerald Amp MAX PCR master mix (Takara, Japan), 0.5 µl of each primer (10 pmol/µl; Metabion, Germany) and completed up to 25 µl by PCR-grade water. The PCR products were electrophoresed on a 1.5% agarose gel and visualized under ultraviolet light. The specific oligonucleotide primers set, and amplification conditions are presented in Table 1. A negative control was included, containing all components of the PCR mixture but with water instead of template DNA. The positive control was the *S. aureus* strain ATCC 700,699.

### Molecular detection of *S. aureus* virulence gene

Phenotypic MDR-MRSA isolates were exposed to uniplex PCR, which targets the toxic shock syndrome toxin gene (*tsst-1*), according to Havaei et al.^47^. The PCR mixtures were carried out on a total volume of 25 µl, containing 4 µl of template DNA from each isolate, 12.5 µl of Emerald Amp MAX PCR master mix (Takara, Japan), 0.5 µl of each primer (10 pmol/µl; Metabion, Germany) and completed up to 25 µl by PCR-grade water. The PCR products were electrophoresed on a 1.5% agarose gel and visualized under ultraviolet light. The specific oligonucleotide primers set, and amplification conditions are demonstrated in Table 1.

### Statistical analysis

Statistical analysis was conducted in R (version 4.2.2, R Foundation for Statistical Computing). The isolates were clustered using the pheatmap library (version 1.0.12)^48^.

## Results

### Prevalence of *S. aureus* among the examined oyster samples

Thirteen confirmed *S. aureus* isolates were detected in 33 fresh oyster pooled samples collected from various retail fish markets in Egypt, resulting in a 39.4% prevalence rate. All phenotypic *S. aureus* isolates tested positive for the 16 S rRNA and *nuc* genes.

### Antimicrobial susceptibility profile of the identified *S. aureus* isolates

**Fig. 1:**
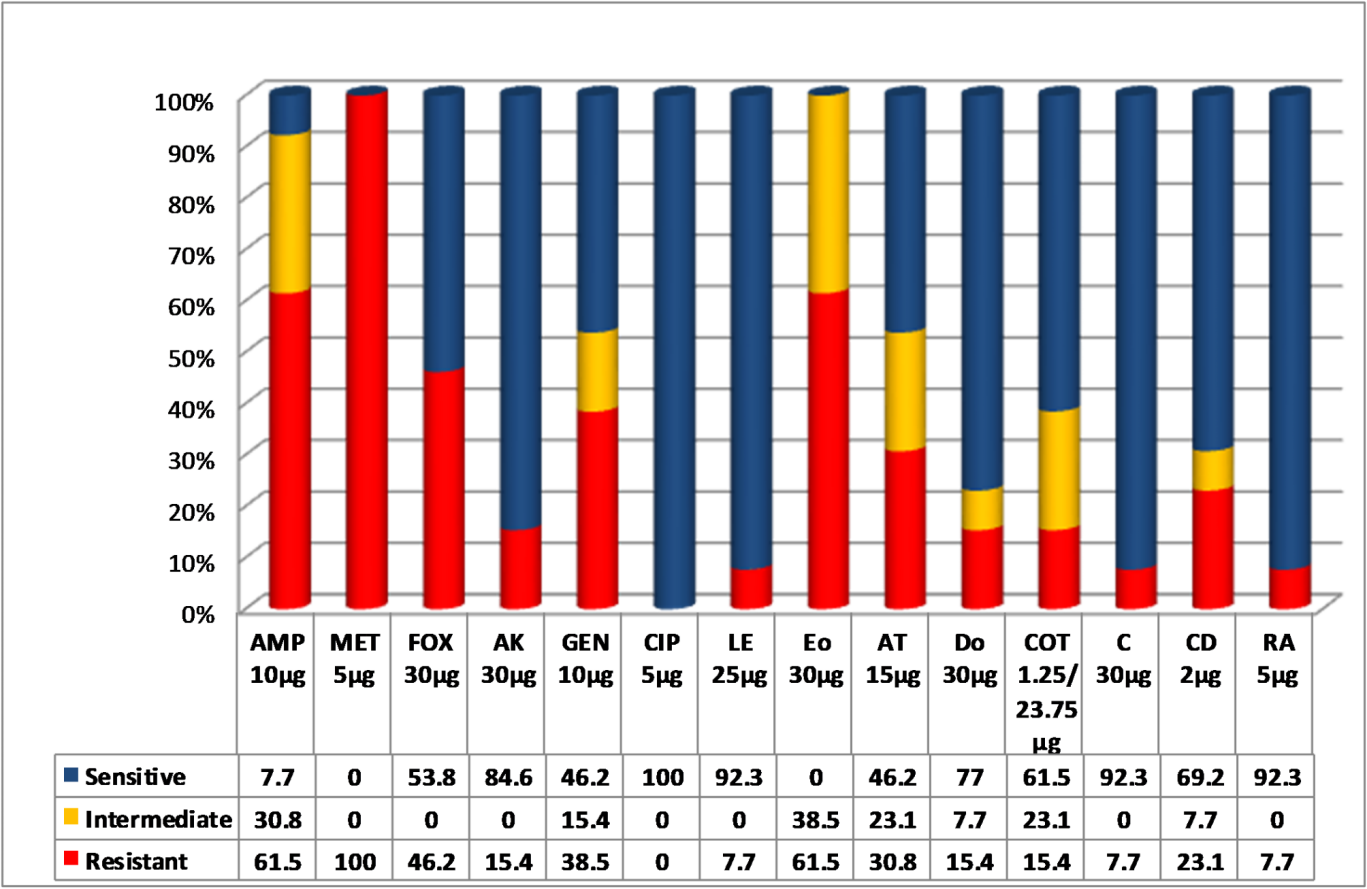
Percentage of antimicrobial resistance, intermediate resistance, and sensitive determined by disc diffusion method in *S. aureus* isolates from retail oysters in Egypt. (AMP, Ampicillin; MET, Methicillin; FOX, Cefoxitin; AK, Amikacin; EN, Gentamycin; CIP, Ciprofloxacin; LE, Levofloxacin; Eo, Erythromycin; AT, Azithromycin; Do, Doxycycline; COT, Trimethoprm-Sulfamethoxazole; C, Chloramphenicol; CD, Clindamycin; RA, Rifampicin).

Our results showed that all the isolates exhibited resistance to more than one of the examined antibiotics, with 100% of isolates showing resistance against methicillin (MET). Whereas, ciprofloxacin (CIP) demonstrated the highest effectiveness, with a 100% susceptibility rate, followed by levofloxacin (LE) and rifampicin (RA), which showed 92.3% susceptibility, with other susceptibility patterns shown in Fig. 1.

**Fig. 2:**
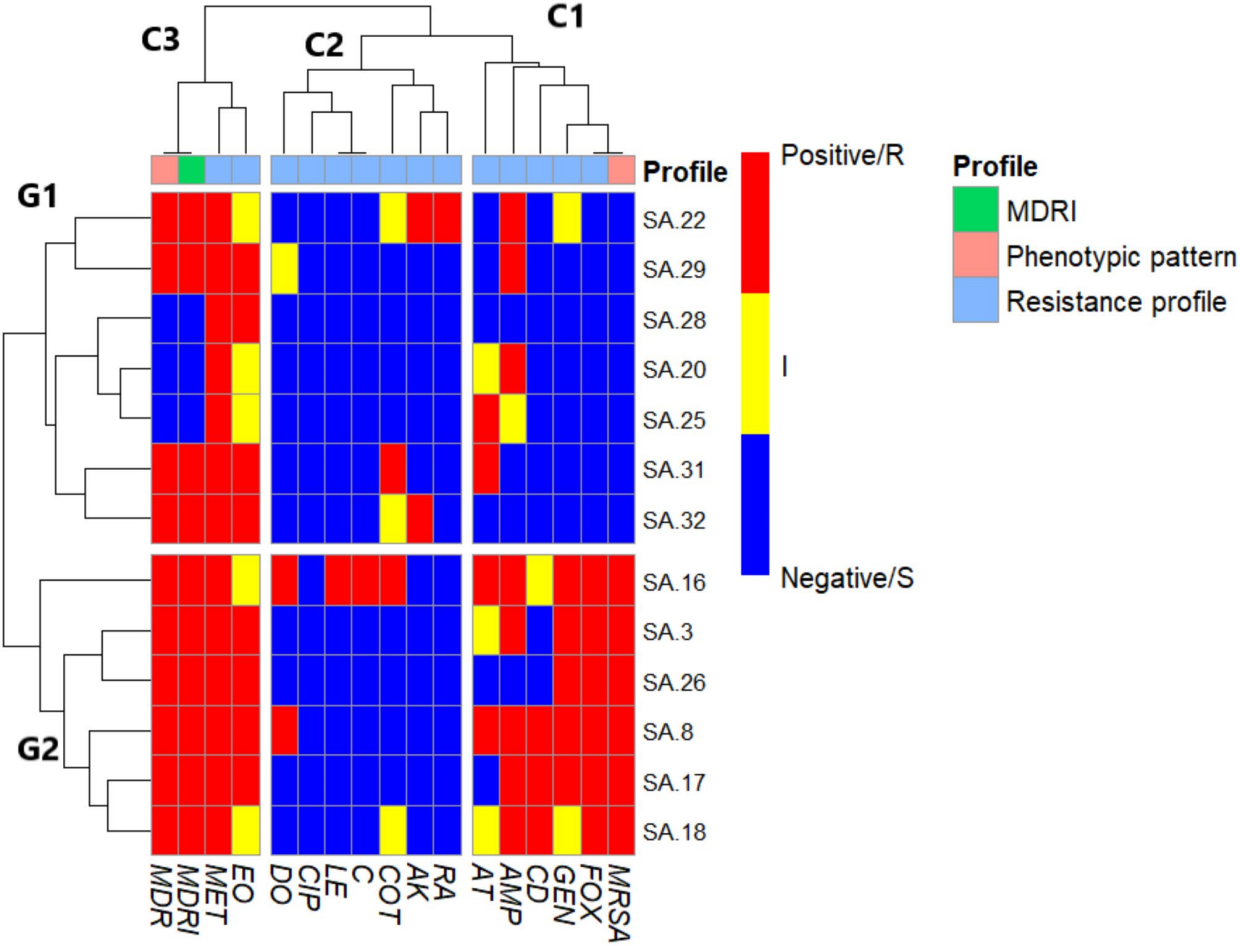
Heatmap of *S. aureus* isolates clarified the resistance profile against fourteen antibiotic discs. The phenotypic pattern (MRSA and MDR) and Multi Drug Resistance Index (MDRI) also identified. The varied colors represent positive MRSA or MDR and MDRI exceeding 0.2 threshold (red) or negative MRSA or MDR and MDRI lower than 0.2 (blue) results; regarding the resistance profile; resistant phenotype (red), intermediate resistant (yellow), and sensitive (blue).

Furthermore, the results of the study revealed that 10 (77%) of the13 isolates were classified as MDR with MARI exceeding 0.2, whereas 3 isolates (23.1%) exhibited a MARI below 0.2. Notably, none of the isolates showed an index of 1.0, as detailed in (Fig. 2 & Supplementary Table 1).

Notably, MRSA isolates were recovered from 6 (46.2%) of the 13 confirmed *S. aureus* isolates based on phenotypic resistance to cefoxitin. All six MRSA isolates exhibited MDR, displaying resistance to both methicillin and cefoxitin. However, one MRSA isolate showed unexpected susceptibility to ampicillin. Among these, four MRSA isolates were not susceptible to clindamycin, and two isolates showed non-susceptibility to trimethoprim-sulfamethoxazole, as shown in Fig. 2 and Supplementary Table 1.

### Occurrence of *S. aureus* resistance and virulence genes

**Fig. 3:**
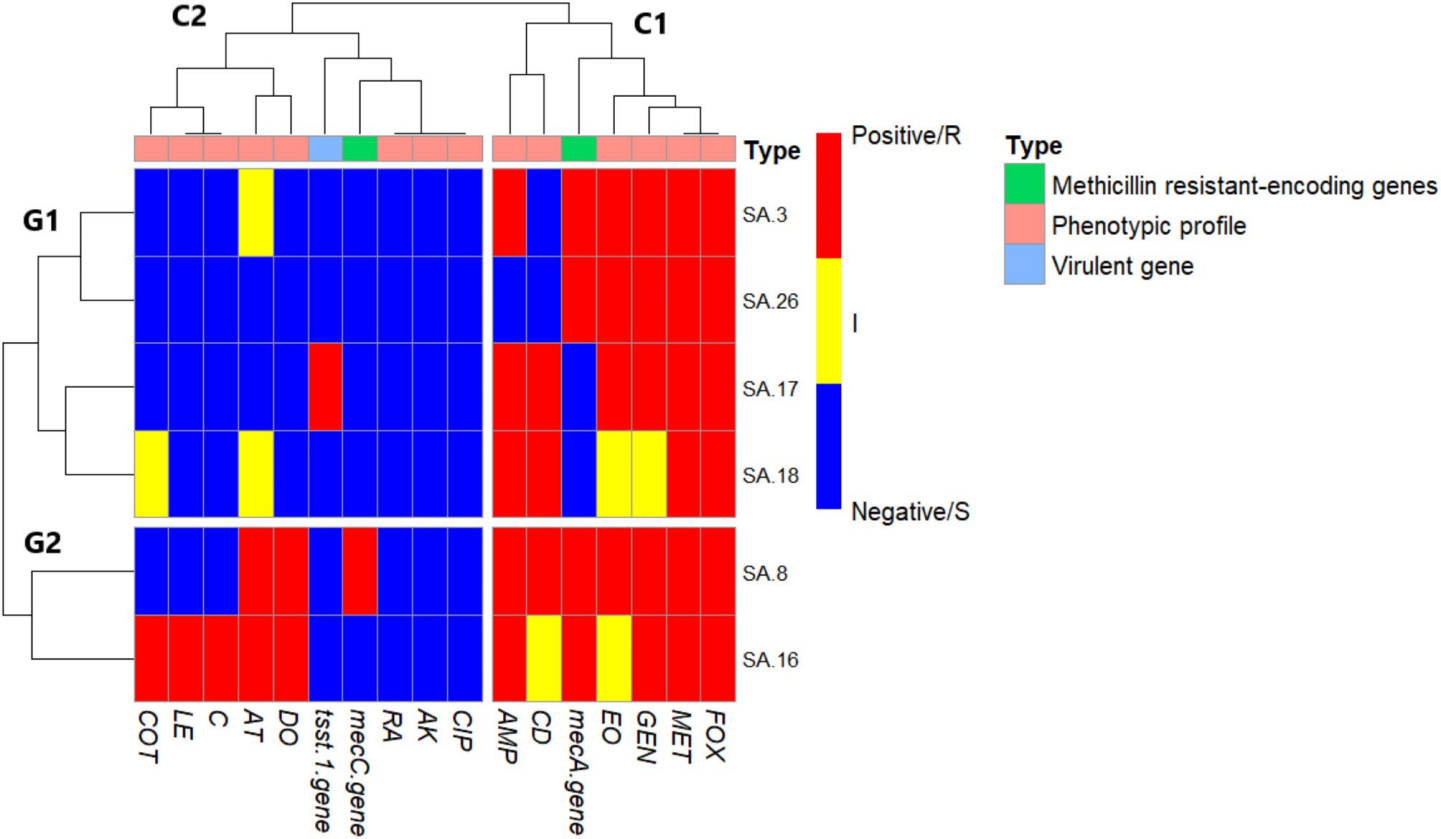
Heatmap of multidrug-resistant-methicillin- resistant *S. aureus* (MDR-MRSA) isolates clustered according to both antimicrobial resistance phenotypes with methicillin resistant (*mecA* & *mecC*) and virulence gene carriage (*tsst-1*). The varied colors represent positive methicillin resistant and virulence gene (red) or negative (blue) results; regarding the antimicrobial resistance; resistant phenotype (Red), intermediate resistant (Yellow), and sensitive (blue).

The PCR results for methicillin resistance-encoding genes, *mecA* and *mecC*, and the toxic shock syndrome toxin (*tsst-1*) in phenotypic MDR-MRSA revealed that 66.7% (4 out of 6) of the isolates harbored the *mecA* gene, while 16.7% (1 out of 6) carried the *mecC* gene. Two MRSA isolates lacked both *mecA* and *mecC* genes. Additionally, the *tsst-1* virulence gene was identified in one isolate (16.7%), as shown in Fig. 3 and Supplementary Table 2.

### Cluster analysis of the antimicrobial sensitivity results (phenotypic and genotypic) with virulence gene carriage in MDR-MRSA isolates (*n* = 6)

The heatmap (Fig. 3) categorizes six MDR-MRSA isolates into two main groups (G1 and G2) based on their susceptibility to 14 tested antibiotics, two genes encoding methicillin-resistance, and one virulence gene. The top of the heat map (C1 and C2) shows the phenotypic resistance profile with methicillin resistance and virulence genes tested.

Within the clustering, two MDR-MRSA isolates (SA.8 and SA.16) had nearly identical resistance profiles, with SA.8 carrying *mecA* and *mecC* genes and SA.16 carrying *mecA* alone. Both isolates were negative for *tsst-1* virulence gene.

## Discussion

Globally, food safety and public health are seriously threatened by the prevalent foodborne bacterium *S. aureus*^6^. In this study, *S. aureus *was detected in 13 out of 33 pooled oyster samples, accounting for 39.4% of the samples. This reflects post-harvest contamination, possibly originating from inadequate storage and poor sanitation in the markets^49,50^. Workers handling oysters without protective clothes may inadvertently transfer *S. aureus *from their throats, nasal passages, or hands^51^. Nevertheless, this does not preclude the possibility that *S. aureus* can naturally occur in oysters. Given that seafood is protein-rich, it provides an ideal environment for the growth of *S. aureus*^52^.

Antibiotic resistance is a growing global issue, and several studies have documented drug-resistant *S. aureus *in seafood^24,30,53,54^. In the current study, 77% (10/13) of the isolates were classified as MDR, with MARI values exceeding 0.2, indicating that the isolates originated from a source where antibiotics were used to a great degree and/or in large amounts^45^.

The prevalence of MRSA infections and colonization in bivalves and other seafood has steadily increased over time^24,55^. A study found that 46.2% (6 out of 13) of *S. aureus *isolates from oysters were MRSA, a result consistent with findings in ready-to-eat shellfish in Nigeria^24^. The widespread and uncontrolled use of beta-lactam antibiotics in aquaculture environments, including those where oysters are harvested, contributes to the emergence of MRSA^56,57^.

Notably, all the MRSA isolates obtained were MDR isolates. The evolution of MRSA isolates into MDR forms is a complex interplay of genetic changes, and selective pressures exerted by exposure to various antibiotics^7,58^. Therefore, MRSA is placed second on the list of bacteria of high priority for research and development of new antibiotics^59^. The emergence of MDR-MRSA in seafood poses a significant public health risk and raises concerns about potential transmission to humans, emphasizing their zoonotic potential^25,60^.

The current study revealed that one MRSA isolate from oysters showed susceptibility to ampicillin. This unexpected result may be attributed to hetero-resistance, a phenomenon where a subpopulation of MRSA bacteria remains susceptible to beta-lactams while the majority is resistant^61^. Additionally, some MRSA isolates exhibit non-susceptibility to clindamycin and trimethoprim-sulfamethoxazole, which are typically used to treat MRSA infections^62,63^. This finding alarms the public health community that MRSA becomes more resistant to advanced antibiotics, which may make it non-curable in the future.

In the context of MRSA genes, the current study found that 66.7% (4 out of 6) of the isolates harbored the *mecA *gene, which is frequently detected and serves as the gold standard for identifying MRSA^64,65^. In contrast, 1 out of 6 isolates (16.7%) carried the *mecC* gene which is aligns with a study conducted in Egypt by Shebl et al.^66^, which identified the *mecC* gene in three MRSA isolates, representing 6% of the total isolates. However, other studies globally have reported that certain MRSA isolates exhibit phenotypic resistance despite the absence of *mecA* or *mecC *genes^67–70^. This resistance is likely due to the presence of alternative genes that confer beta-lactam resistance in MRSA^71^. Additionally, genetic mutations that alter the target site of beta-lactam antibiotics and the overproduction of β-lactamase enzymes are significant contributing factors^72,73^. To ensure comprehensive detection, combining both genotypic and phenotypic methods is advisable, minimizing the risk of missing genetically divergent strains.

*S. aureus* produces a remarkable range of virulence factors that facilitate their pathogenicity. The current study is the first of its kind to identify the toxic shock syndrome toxin gene (*tsst-1*) in one out of six (16.7%) MDR- MRSA isolates found in Egyptian oysters. This finding highlights the heightened toxicity associated with this strain, potentially endangering both seafood handlers and public consumers since they may acquire this virulence gene through the food chain, leading to serious diseases with limited treatment options^74–76^.

In the investigation of pathogen diversity and evolution, MDR-MRSA isolates were clustered based on their antimicrobial resistance phenotypes with methicillin-resistant (*mecA* & *mecC*) and virulence gene carriage (*tsst-1*) using the pheatmap library. Notably, isolates (SA.8 and SA.16) displayed nearly identical resistance and virulence profiles. This similarity suggests a common source, possibly contaminated water, handling practices, or processing facilities. Additionally, these isolates may share common suppliers or geographic origins, contributing to similar microbial populations, aligning with findings from studies by Chen et al.^77^ and Yu et al.^78^.

## Conclusion

The study highlights significant epidemiological concerns by identifying the prevalence of MDR-MRSA in retail oysters in Egypt, raising critical public health and food safety issues. These findings enhance our understanding of the development and dissemination of antibiotic resistance within aquatic ecosystems. Furthermore, the study emphasizes the need for enhanced sanitary education for food handlers, who may act as reservoirs and vectors for MRSA. Future investigations employing larger sample sizes and advanced genomic methodologies are essential to deepen insights into this pressing issue.

## Electronic supplementary material

Below is the link to the electronic supplementary material.

Supplementary Material 1

## Data Availability

All the data generated or analyzed in this study are included in this published article.
