# Supplementary material for: Public health implications of multidrug-resistant and methicillin-resistant Staphylococcus aureus in retail oysters
Source: Sci Rep. 2025 Feb 6;15:4496. doi: 10.1038/s41598-025-88743-5 (PMC11802730; doi:10.1038/s41598-025-88743-5)
Supplement: Supplementary file 1 — Supplementary Material 1 [file 41598_2025_88743_MOESM1_ESM.docx]

| ***S. aureus* isolates** | **Antibiogram profile** | | | | | | | | | | | | | | **MRSA** | **MDR** | **MDRI** |
| --- | --- | --- | --- | --- | --- | --- | --- | --- | --- | --- | --- | --- | --- | --- | --- | --- | --- |
|  | **AMP** | **MET** | **FOX** | **AK** | **GEN** | **CIP** | **LE** | **EO** | **AT** | **DO** | **COT** | **C** | **CD** | **RA** |  |  |  |
| **SA. 3** | R | R | R | S | R | S | S | R | I | S | S | S | S | S | MRSA | MDR | 0.35 |
| **SA. 8** | R | R | R | S | R | S | S | R | R | R | S | S | R | S | MRSA | MDR | 0.57 |
| **SA. 16** | R | R | R | S | R | S | R | I | R | R | R | R | I | S | MRSA | MDR | 0.64 |
| **SA. 17** | R | R | R | S | R | S | S | R | S | S | S | S | R | S | MRSA | MDR | 0.42 |
| **SA. 18** | R | R | R | S | I | S | S | I | I | S | I | S | R | S | MRSA | MDR | 0.28 |
| **SA. 20** | R | R | S | S | S | S | S | I | I | S | S | S | S | S | - | - | 0.14 |
| **SA. 22** | R | R | S | R | I | S | S | I | S | S | I | S | S | R | - | MDR | 0.28 |
| **SA. 25** | I | R | S | S | S | S | S | I | R | S | S | S | S | S | - | - | 0.14 |
| **SA. 26** | S | R | R | S | R | S | S | R | S | S | S | S | S | S | MRSA | MDR | 0.28 |
| **SA. 28** | S | R | S | S | S | S | S | R | S | S | S | S | S | S | - | - | 0.14 |
| **SA. 29** | R | R | S | S | S | S | S | R | S | I | S | S | S | S | - | MDR | 0.21 |
| **SA. 31** | S | R | S | S | S | S | S | R | R | S | R | S | S | S | - | MDR | 0.28 |
| **SA. 32** | S | R | S | R | S | S | S | R | S | S | I | S | S | S | - | MDR | 0.21 |
| **Total (%)** | | | | | | | | | | | | | | | **6 (46.2)** | **10 (77)** |  |

**Table S 1**. Antimicrobial susceptibility profiles and MDR Index of the identified *S. aureus* isolates

***R, I, and S**, represent Resistance, Intermediate resistance and sensitive isolates, respectively.

***MARI** =$\frac{\begin{aligned} Number of antibiotics to whichh the isolate is resistant \\ \end{aligned}}{The total number of antibiotics tested}$

| **MDR-MRSA isolates** | **Phenotypic profile** | | | | | | | | | | | | | | **Genotypic profile** | | |
| --- | --- | --- | --- | --- | --- | --- | --- | --- | --- | --- | --- | --- | --- | --- | --- | --- | --- |
|  |  |  |  |  |  |  |  |  |  |  |  |  |  |  | **Methicillin resistant-encoding genes** | | **virulence gene** |
|  | **AMP** | **MET** | **FOX** | **AK** | **GEN** | **CIP** | **LE** | **EO** | **AT** | **DO** | **COT** | **C** | **CD** | **RA** | ***mecA* gene** | ***mecC* gene** | ***tsst-1* gene** |
| **SA.3** | R | R | R | S | R | S | S | R | I | S | S | S | S | S | + | _ | _ |
| **SA.8** | R | R | R | S | R | S | S | R | R | R | S | S | R | S | + | + | _ |
| **SA.16** | R | R | R | S | R | S | R | I | R | R | R | R | I | S | + | _ | _ |
| **SA.17** | R | R | R | S | R | S | S | R | S | S | S | S | R | S | _ | _ | + |
| **SA.18** | R | R | R | S | I | S | S | I | I | S | I | S | R | S | _ | _ | _ |
| **SA.26** | S | R | R | S | R | S | S | R | S | S | S | S | S | S | + | _ | _ |

**Table S2.** Occurrence of methicillin encoding *mecA* and *mecC* genes and *tsst-1* virulence gene among multidrug-resistant methicillin resistant *S. aureus* isolates.
